# Prevalence of ESBL-Producing *Klebsiella pneumoniae* Isolates in Tertiary Care Hospital

**DOI:** 10.5402/2011/318348

**Published:** 2011-12-01

**Authors:** Vemula Sarojamma, Vadde Ramakrishna

**Affiliations:** ^1^Department of Microbiology, Government Medical College, Anantapur 515001, India; ^2^Department of Biotechnology and Bioinformatics, Yogi Vemana University, Kadapa 516003, India

## Abstract

Extended-spectrum **β** lactamases (ESBLs) continue to be a major challenge in clinical setups world over, conferring resistance to the expanded-spectrum cephalosporins. An attempt was made to study the prevalence of ESBL-producing *Klebsiella pneumoniae* clinical isolates in a tertiary care hospital in Kurnool. A total of hundred collected isolates of *Klebsiella pneumoniae* was studied for their susceptibility patterns to various antibiotics and detection of ESBL producers by double disc synergy test (DDST) and phenotypic confirmatory disc diffusion test (PCDDT). Of the 100 isolates tested for their antibiogram, 61% isolates have shown susceptibility to 3rd-generation cepholosporins and 39% were resistant. Amoxycillin showed the highest percentage of resistance followed by tetracyclins and cotrimoxazole. Among 39 resistant isolates of *Klebsiella pneumoniae*, 17 were ESBL producers detected by DDST and PCDDT. ESBL producers were more in the hospital isolates (28%) compared to community isolates (6%). Maximum percentage of ESBL producers were noticed from blood sample with 57.14%. In the present study, a large number of isolates were found to be multidrug resistant and ESBL producers. PCDDT was found to be better than DDST in the detection of ESBLs. Continued monitoring of drug resistance is necessary in clinical settings for proper disease management.

## 1. Introduction

*β*-Lactam antimicrobial agents represent the most common treatment for bacterial infections and continue to be the leading cause of resistance to *β*-lactam antibiotics among Gram-negative bacteria worldwide. The persistent exposure of bacterial strains to a multitude of *β*-lactams has induced dynamic and continuous production and mutation of *β*-lactamases in these bacteria, expanding their activity even against the newly developed *β*-lactam antibiotics. These enzymes are known as extended-spectrum *β*-lactamases (ESBLs) [1–3]. Treatment of these multiple drug-resistant organisms is a therapeutic challenge. At the level of a wider geographic scale, the incidence of ESBL-producing organisms is difficult to determine due to various reasons, difficulty in detecting ESBL production and inconsistencies in reporting [4]. In recent surveys, a significant increase in the ESBL rate was reported from all parts of the world [5–12]. 

*Klebsiella pneumoniae *and* Escherichia coli *remain the major ESBL-producing organisms isolated worldwide [13] which are recommended to be routinely tested for and reported by the Clinical and Laboratory Standards Institute [14]. Prevalence of ESBLs varies from an institute to another. Previous studies from India and abroad have reported ESBL production varying from 8 to 80%. However, there is paucity scientific information available on antibiotic profile with rate of ESBL production in *Klebsiella pneumoniae* isolates. Keeping in view the above facts, the present study was undertaken to find the prevalence of ESBL producers among *Klebsiella pneumoniae* isolates at our institute.

Government General Hospital, Kurnool is a 1200-bedded hospital with a daily outpatient turnover of more than 2000. It is a tertiary care hospital and a referral centre to the hospitals in the surrounding districts. *Klebsiella pneumoniae* is one of the commonest organisms associated with the hospital acquired infections. Hence, the present study is attempted to evaluate the extent of prevalence of ESBL-producing strains of *Klebsiella pneuoniae* in the hospital and community along with antibiotic resistance profile.

## 2. Materials and Methods

This prospective study was conducted in the Department of Microbiology at Kurnool Medical College, Kurnool, Andhra Pradesh. A total of 100 isolates were obtained from clinical samples from January to October 2008. Among 100 isolates, 50 were from out patients and remaining 50 from in patients admitted into Government General Hospital in units like medical, surgical, orthopedic, burns, pediatric, neonatal intensive care units, and acute medical care units.

## 3. Processing of Samples

All samples were inoculated on Mac Conkey's and Blood agar, incubated at 37°C for overnight, and colonies were processed. In case of blood sample, blood was incubated at 37°C overnight in Brain Heart infusion broth. A drop of Brain Heart Infusion broth was inoculated on Mac Conkey agar and Blood agar and incubated at 37°C for overnight. If colonies were not seen even after 7 days, the blood sample was reported as negative. Colonies in any of these 7 days, were subcultured. *Klebsiella pneumoniae* isolates that were obtained as a pure and predominant growth from the clinical specimens were only considered for the present study. The organisms were identified based on colony morphology and biochemical reactions [15].

## 4. Antimicrobial Susceptibility Testing

Routine disc diffusion susceptibility testing was performed by modified KirbyBauer's disc diffusion method [16]. Various antimicrobial discs were used which include antimicrobials for screening of ESBL *Klebsiella *species: cefotaxime—30 *μ*g; ceftazidime—30 *μ*g; ceftriaxone—30 *μ*g; amikacin—10 *μ*g; amoxycillin—20 *μ*g; gentamycin—10 *μ*g; tetracycline—30 *μ*g; imipenem—30 *μ*g; ciprofloxacin—5 *μ*g; aztreonam—30 *μ*g; cotrimoxazole—1.25/23.75 *μ*g. The results were interpreted as per the National Committee for Clinical Laboratory Standards (NCCLS) recommendations [17]. *Klebsiella pneumoniae* ATCC 700 603 (ESBL positive) strain was used as control throughout the study. Isolates with resistance or with decreased susceptibility (intermediate by NCCLS criteria) to any of the 3GC were selected for further study.

## 5. Screening of ESBL-Producing Strains for *Klebsiella pneumoniae*

Clinical and Laboratory Standards Institute [14] has developed screening tests for identifying the ESBL-producing *Klebsiellla* species. According to CLSI guidelines, strains showing zone of inhibition of ≤22 mm for ceftazidime, ≤27 mm for cefotaxime, and ≤25 mm for ceftriaxone were selected for conformational tests of ESBL.

### 5.1. ESBL Confirmatory Tests

#### 5.1.1. Double Disc Synergy Test (DDST) [18]

The isolated colonies were inoculated in peptone water at 37°C for 2–6 h. The turbidity was adjusted to 0.5 Mc Farlands standard and lawn culture was made on Mueller-Hinton agar using sterile swab. Augmentin disc (20/10 *μ*g) was placed in the centre of plate. Both side of Augmentin disc, a disc of cefotaxime (30 *μ*g) and ceftazidime (30 *μ*g), were placed with centre to centre distance of 15 mm to centrally placed disc. The plate was incubated at 37°C overnight. ESBL production was interpretated as the 3^rd^-generation cephalosporin disc, inhibition was increased towards the Augmentin disc or if neither discs were inhibitory alone but bacterial growth was inhibited where the two antibiotics were diffused together.

#### 5.1.2. Phenotypic Confirmatory Disc Diffusion Test (PCDDT) for ESBL [17]

ESBL production was confirmed among potential ESBL-producing isolates by phenotypic tests. Lawn culture of the organism was made and 3^rd^-generation cephalosporins ceftazidime (30 *μ*g) disc and ceftazidime + clavulinic acid (30 *μ*g + 10 *μ*g) disc was placed with 25 mm apart. An increase of ≥5 mm in zone of inhibition for ceftazidime + clavulinic acid compared to ceftazidime was confirmed as ESBL producers.

## 6. Results

A significant difference in resistant and susceptibility pattern was observed with 3rd-generation cepolosporins between hospital and community strains. An antibiogram of the isolates was presented in (Table 1). Of the 100 isolates of *Klebsiella pneumoniae* tested for their antibiogram, 61% isolates have shown susceptibility to 3rd-generation cepholosporins and 39% were resistant. Amoxycillin showed the highest percentage of resistance (86% in hospital and 76% in community) followed by tetracyclins and cotrimoxazole. Similarly, a highest percentage susceptibility to imipenam (84% hospital, 96% community) followed by ceftriaxone, cefaperazone + sulbactum were noticed. In the case of aminoglycosides, amikacin showed higher percentage of susceptibility (56% in hospital and 78% in community) compared to gentamycin (40% in hospital and 62% in community).

 Of the 100 clinical isolates of *Klebsiella pneumoniae*, 39 were screened according to CLSI guidelines and selected for conformational tests of ESBL. The two techniques were used in the present study to confirm ESBL-producing *Klebsiella pneumoniae,* namely, DDST and PCDDT and confirmed 28% hospital and 6% community isolates were the ESBL producers. The results (Table 2) showing that of the 17 *Klebsiella pneumoniae* isolates, 15 isolates were positive by DDST (88.23%), however, PCDDT shown that all 17 were positive (100%). Among 17 strains of ESBL-producing *Klebsiella pneumoniae*, 3 isolates are resistant to one of the 3GCs, 5 are against the 2 of the 3GCs, and 9 are resistant to all the 3GCs. DDST fail to detect ESBL in 2 isolates which showed ESBL production by PCDDT. There is no instance of a DDST-positive and PCDDT-negative ESBL producers. This implies that PCDDT is more sensitive in detecting ESBL production than DDST. Among 17 confirmed ESBL producers, the Hospital isolates are the major ESBL producers.

## 7. Discussion

In India, high prevalence of ESBL-producing *Klebsiella pneumoniae *strains has been reported by various groups [19–22]. In the present study, we noticed the prevalence of ESBL-producing *Klebsiella* is 17%. The percentage of ESBL-producing organisms ranged from 4% to 83% in India. The percentage is lowest in Maharastra reported by Rodrigues et al. [21] that the 4 (8.5%) positive ESBL producers among 47 *Klebsiella pneumoniae* isolates. Probably, it reflects emerging phase of ESBL production which by now would have increased in the same place if a similar study is conducted in the present time. This is understandable as the prevalence of ESBL producers in any hospital depends upon various factors like antibiotic policy, the carriage rate among the hospital personal, and the type of disinfection used especially in ICU [23]. These have not been extensive studies available in India. These strains are often undetectable by routine susceptibility testing methods. It can be presumed that ESBL-producing strains are more prevalent than currently recognized.

In the present study, among the 50 nosocomial isolates, 30 isolates were shown resistance to 3GCs (60%). Of these 30 isolates, the 14 showed ESBL production (28%). However, the percentage of ESBL producers in community isolates tested was only 6%. The present results were correlated with that of Shukla et al. [24] reported that 32% of *Klebsiella pneumoniae* from 120 samples of tertiary care hospital. Some of the authors feel that ESBL screening is not likely to affect the patient outcome and hence neither necessary nor cost, effective for the laboratories. They also observed good clinical outcome with cephalosporins for treatment of infections with ESBL-producing organisms. This is an argument against routine screening for ESBL production [25, 26]. 

In the present study, the highest percentage of ESBL was reported from blood followed by stool, sputum, urine, and pus samples (Table 3). A total number of 7 blood samples were processed from septicemic patients and reported 57.14% samples to have ESBL strains of *Klebsiella pneumoniae.* Similar reports have been recorded in the recent past by Guptha et al. [27]: from Chandigarh isolated 9 ESBL-producing *Klebsiella pneumoniae* from 13 blood samples in septicemia patients with the percentage of 69.2%. Another study by Ananthan and Subha [23] has shown 92.85% ESBL producers from septicemia in a study of 14 cases. The prevalence of ESBL in septicemia individual has great importance as most of ESBL *Klebsilla pneumoniae* are multidrug resistant. There will be great limitation over the choice of drug for treating the septicemia patients. There is 50% incidence of ESBL production in isolates from stool samples. Only two *Klebsiella pneumoniae* isolates could be obtained from stool samples of admitted patients. Here the sample number is quite less and hence percentage does not depict the exact one. However, the gastrointestinal carriage and asymptomatic colonization with ESBL producers among inpatients has been well documented [28].

A significant number of ESBL-positive cases are recorded from sputum samples. Out of 42 sputum samples 8 were ESBL positive with the percentage of 19.04%. This may be because many of the sputum samples are taken from ICU wards. In the present study, 28% ESBLs are reported from patients admitted into hospital. A study conducted in Aligarh tertiary care hospital has also reported 30.18% ESBL *Klebsiella pneumonaie* from clinical samples [27]. A study conducted by Ananthan and Subha [23] from Chennai reported 23.6% of ESBL *Klebsiella pneumoniae* from clinical isolates. In other studies, Menon et al. [29] from Chennai and Supriya et al. [30] from Nagpur have also reported the prevalence of ESBL producing *Klebsiella pneumoniae *were 21.2% and 25.65%, respectively.

In recent years, a significant increase in ESBL-producing *Klebsiella *spp. was also reported from USA 4.2–44% [30–32] Canada 4.9% [33], Spain 20.8% [9], Taiwan 28.4% [10], Turkey 78.6% [11], Algeria 20% [12], and China 51% [34]. Focusing on the epidemiology in Europe, there are considerable geographical differences in the occurrence of ESBLs. A recent large survey of 1610 *Escherichia coli *and 785 *K. pneumoniae *isolates from 31 centers in 10 European countries found that the prevalence of ESBL in these organisms ranged from as low as 1.5% in Germany to as high as 39–47% in Russia, Poland, and Turkey [35].

Looking at the overall trend of ESBL *Klebsiella pneumoniae* is on the rise and variable. This could partly be irrational use of cephalosporins at some institutions and more number of blood samples was processed. The actual magnitude of problem posed by ESBL producers is not known as routine susceptibility testing fails to detect all ESBL producers. The two techniques used in the present study to confirm ESBL production are, namely, DDST and PCDDT. DDST fail to detect ESBL in 2 isolates which showed ESBL production by PCDDT. There is no instance of a DDST-positive and PCDDT-negative ESBL producers. This implies that PCDDT is more sensitive in detecting ESBL production than DDST. Looking at all other authors and in the present study, it is confirmed that PCDDT is more sensitive than DDST for detection of ESBLs [36–38].

In conclusion, our study highlights the prevalence of ESBL-producing *Klebsiella pneumoniae* in Government General Hospital, Kurnool, having a significant percentage. Routine detection of ESBL-producing microorganisms is required to be done by each laboratory by the standard detection methods so as to control the spread of these infections and also to institute proper therapeutic strategies. For the detection, the phenotypic confirmatory disc diffusion test is simple, sensitive, and costeffective. However there is a need to emphasize on the rational use of antimicrobials and strictly adhere to the concept of “reserve drugs” to minimize the misuse of available antimicrobials. In addition, regular antimicrobial susceptibility surveillance is essential.

## Figures and Tables

**Table 1:** Antibiogram of clinical isolates of *Klebsiella pneumoniae. *

S. no.	Name of antibiotic	Hospital isolates				Community isolates			
		Susceptibility		Resistant		Susceptibility		Resistant	
		No. of isolates	Percentage	No. of isolates	Percentage	No. of isolates	Percentage	No. of isolates	Percentage
(1)	Amikacin	28	56%	22	44%	39	78%	11	22%
(2)	Gentamycin	20	40%	30	60%	23	62%	27	38%
(3)	Ciprofloxacin	14	28%	36	72%	19	38%	31	62%
(4)	Amoxycillin	7	14%	43	86%	12	24%	38	76%
(5)	Ceftazidime	22	44%	28	56%	40	80%	10	20%
(6)	Ceftriaxone	28	56%	22	44%	42	84%	8	16%
(7)	Cefotaxime	26	52%	24	48%	39	78%	11	22%
(8)	Cotrimoxazole	10	20%	40	80%	13	26%	37	74%
(9)	Imipenem	42	84%	8	16%	48	96%	2	9%
(10)	Aztreonam	15	30%	35	70%	14	28%	36	72%
(11)	Cefaperazone + Sulbactum	30	62%	20	38%	40	82%	10	18%
(12)	Tetracyclin	4	8%	42	84%	12	24%	38	76%

**Table 2:** ESBL pattern of *Klebsiella pneumoniae* isolated from various clinical samples.

No. of *Klebsiella pneumoniae* isolates	Hospital isolates	Community isolates	No. of screened and selected isolates for ESBL confirmatory tests		No. of ESBL confirmed		No. of ESBL confirmed by	
			(39)		(17)			
			Hospital	Community	Hospital	Community	DDST	PCDDT
100	50	50	30	9	14 (28%)	3 (6%)	15 (88%)	17 (100%)

**Table 3:** Sample wise distribution of ESBL producing *Klebsiella penumoniae. *

S. no.	Sample	No. *Klebsiella pneumoniae* isolates	ESBL producing *K. pneumoniae *	
			No. of isolates	Percentage
1	Sputum	42	8	19.04%
2	Urine	23	3	13.04%
3	Pus	16	2	12.05%
4	Blood	7	4	57.14%
5	Stool	2	1	50%
